# The financial performance of private hospitals in Saudi Arabia: An investigation into the role of internal control and financial accountability

**DOI:** 10.1371/journal.pone.0285813

**Published:** 2023-05-22

**Authors:** Naseem Al Rahhaleh, Tawfeek A. Al-khyal, Abdullah Daghran Alahmari, Mohammed Khaled Al-Hanawi

**Affiliations:** 1 Faculty of Economics and Administration, Finance Department, King Abdulaziz University, Jeddah, Saudi Arabia; 2 Faculty of Economics and Administration, Accounting Department, King Abdulaziz University, Jeddah, Saudi Arabia; 3 Directorate of Health Affairs in Jeddah Governorate, Ministry of Health, Jeddah, Saudi Arabia; 4 Faculty of Economics and Administration, Department of Health Services and Hospital Administration, King Abdulaziz University, Jeddah, Saudi Arabia; 5 Health Economics Research Group, King Abdulaziz University, Jeddah, Saudi Arabia; University of Murcia: Universidad de Murcia, SPAIN

## Abstract

The aim of this study was to examine the relationship between internal control, financial accountability, and financial performance in the private healthcare sector in the Kingdom of Saudi Arabia (KSA) through a questionnaire survey of 78 private hospitals. Drawing on agency theory, the study utilized structural equation modelling with partial least-squares technique to test multiple hypotheses. Results indicate a significant positive relationship between internal control and financial performance, with financial accountability acting as a mediator. Additionally, financial accountability was found to have a direct positive effect on financial performance. These findings provide new evidence for improving financial performance in private hospitals in the KSA through the implementation of internal control and financial accountability measures. Further research could examine additional factors that may impact financial performance in the healthcare sector.

## Introduction

The financial performance of firms and institutions remains a topic of global interest given challenging widespread phenomena such as market crashes, fiscal mismanagement, and corrupt activities, including fraud [1]. The global financial crisis of 2008 and the ongoing economic effects of the COVID-19 pandemic have increased the urgency for organizations to scrutinize their financial practices. As globalization has expanded, financial mistakes can result in complex consequences, including firm closures [2]. Public and private institutions have struggled to achieve their objectives in the face of global economic challenges [3]. To address this issue, researchers have focused on identifying steps that organizations can take to improve their financial performance, with several studies emphasizing the significance of internal control and financial accountability [4–7].

Organizations utilize internal controls to tackle the agency problem and reduce information asymmetry. As per agency theory [8], information asymmetry creates a principal-agent relationship between contractual parties, where the better-informed party may engage in opportunistic behavior [9]. To address this issue, incentive contracts are commonly employed to align the interests of agents and principals and monitor agents’ efforts. Internal control systems represent an approach that can be used to achieve alignment between an organization’s management and employees, with a view to enhancing financial accountability and performance [10].

This study investigates the relationships that exist between internal control, financial accountability, and financial performance in the private healthcare sector in the Kingdom of Saudi Arabia (KSA). The study is significant given that internal control plays a crucial role in enabling organizations to utilize their resources effectively and efficiently towards achieving their goals [8]. Therefore, implementing and maintaining an internal control system is a prerequisite for enhancing organizational performance [9]. Financial accountability is another critical aspect of organizational performance, as it involves disclosing an organization’s financial activities to stakeholders [10]. Financial accountability emphasizes the responsibility for meeting goals and objectives, and supports sound decision-making. Evaluating financial performance is also essential for organizations as it helps them assess their ability to generate revenue, manage costs, and achieve profitability [11].

This study investigates the relationships that exist between internal control, financial accountability, and financial performance in the private healthcare sector in the Kingdom of Saudi Arabia (KSA). The study is significant given that internal control plays a crucial role in enabling organizations to utilize their resources effectively and efficiently towards achieving their goals [12]. Therefore, implementing and maintaining an internal control system is a prerequisite for enhancing organizational performance [13]. Financial accountability is another critical aspect of organizational performance, as it involves disclosing an organization’s financial activities to stakeholders [14]. Financial accountability emphasizes the responsibility for meeting goals and objectives and supports sound decision-making. Evaluating financial performance is also essential for organizations as it helps them assess their ability to generate revenue, manage costs, and achieve profitability [11].

In the KSA, a high-income country, the healthcare services are provided through the public sector including the Ministry of Health (MOH), other government sector, and the private sector. The MOH is the major public sector provider in the KSA, which delivers healthcare services healthcare services to Saudi citizens free of charge at the point of use, operates a significant number of hospitals and primary healthcare centers. Other government sector, such as security forces medical services, and army forces medical services, provide healthcare services to a defined population, usually their employees and their dependants. In addition, they provide healthcare services to all residents during crises and emergencies. Private hospitals offering healthcare services based on a fee for services paid by the patient as out of pocket or through private health insurance plans [15]. The healthcare system in the KSA represents a significant portion of the national budget and is vulnerable to fluctuations in the unpredictable oil market [16]. The country’s public healthcare system is overburdened due to increased demand since it is free at the point of delivery [17–19]. To meet the demands of healthcare services in the country, the government is considering expanding the role of private healthcare sector [20–22]. Given the government’s policy direction, studying internal control, financial accountability, and financial performance in the private healthcare sector in the KSA is crucial.

Numerous studies have examined internal control, financial accountability, and financial performance, but most of them have focused on public organizations [23–26], non-governmental organizations [11,27], and commercial organizations [1,2,5,28,29]. This study, therefore, contributes to the literature by taking a multidimensional perspective on internal control and addressing the following questions: Does internal control influence financial performance? Does internal control influence financial accountability? Does financial accountability influence financial performance? Furthermore, does financial accountability mediate the influence of internal control on financial performance? Besides, the current literature primarily focuses on internal control in the context of quality-of-service delivery, with limited attention given to financial accountability and performance [24,29,30]. Consequently, this study aims to fill this gap by examining the interplay between internal control, financial accountability, and financial performance in the private healthcare sector in the KSA.

The study employs confirmatory factor analysis (CFA) and structural equation modelling (SEM) using primary data collected from private hospitals in KSA to quantitatively investigate the relationships between internal control and financial accountability, internal control and financial performance, and financial accountability and financial performance. Specifically, partial least-squares (PLS)-SEM approach is utilized as it provides more flexibility in terms of data requirements, model complexity, and relationship specifications [31,32]. This study is timely, considering the significant proportion of the national budget dedicated to healthcare in KSA, and the aim to expand and improve private healthcare provision in line with the Kingdom’s Vision 2030 [21].

## Literature review and hypotheses

Previous studies on internal control systems and financial accountability have drawn upon various theories, including agency theory, attribution theory, contingency theory, risk management theory, and modern portfolio theory. Nyumoo [33] investigated the impact of internal control on the financial performance of Savings and Credit Cooperative Societies in Kenya, utilizing three theories including agency theory, attribution theory, and contingency theory. Using agency theory as a framework, Musah et al. [34] examined the effect of the primary component of an internal control system on the financial performance of small and medium-sized enterprises in Ghana, which constitute approximately 90% of the country’s private sector businesses. Agency theory has also been applied to studies on internal and management audits, such as Tetteh et al.’s [10] research on listed entities on the Ghana stock exchange, where information technology was a moderating variable. Additionally, several studies employed agency theory to explicate how management control systems function and how internal control systems can mitigate information asymmetry and align agents’ interests with those of the principal [35–37].

This study draws upon the agency theory to investigate the relationships that exist between internal control, financial accountability, and financial performance in the private healthcare sector in the KSA. According to agency theory, there exists an information asymmetry between contractual parties [9]. This asymmetry can lead to opportunistic behaviour due to one party having more information than the other. In the principal-agent relationship, the agent has an obligation to the principal, but also has a duty to their own interests [38]. Since the principal cannot fully observe the agent’s effort level, the agent may not be motivated to maximize the benefits for the principal. The agent’s effort level is determined by various factors, including physical effort, pace of work, choice and quality of activities, and search and use of knowledge [39]. The agent is likely to strike a balance between pursuing their own interests and those of the principal. Incentive contracts and monitoring of agents’ efforts are crucial in aligning the interests of agents and principals, as emphasized by agency theory.

In this study, the agency theory is applied to private hospitals where management serves as the principal and staff as the agents. The theory suggests that to achieve financial performance, the organization must minimize the negative impact of the agent-principal relationship by implementing internal control mechanisms. Internal control is defined by the Committee of Sponsoring Organizations of the Treadway Commission as a process that provides reasonable assurance that an organization achieves its operational, reporting, and compliance objectives [12]. It involves the efforts of management and staff aimed at fulfilling an organization’s objectives, and includes policies and procedures designed to ensure that the organization meets the objectives set by its board of directors and executive officers [6]. In essence, internal control refers to an organization’s system of checks and balances [40].

Internal control is a comprehensive process that encompasses control activities, risk assessment, information and communication, and monitoring and evaluation, all of which have an impact on every aspect of an organization’s operations, including administrative, financial, and accounting activities [41]. Control activities are processes, systems, and actions that help implement management directives [10,42]. They impact all operational components of an organization, including all levels and functions [43]. Systematically documenting procedural rules and regulations in this area helps auditors evaluate a firm’s control environment and activities [36,37,44].

Risk assessment refers to the methods and procedures developed by an organization to address various risks that threaten the achievement of its objectives [10,43,45]. It helps prioritize specific goals that have a significant impact on the company’s control systems [36,46,47]. Chen et al. [45] argued that risk assessment enables the discovery of relevant risks that affect the accomplishment of management goals. It involves recognizing and evaluating risks that impact the creation and presentation of financial statements according to the true and fair doctrine and relevant accounting standards [48]. By identifying potential threats to the integrity of the financial reporting system, risk assessment enables management to take preventive measures [49]. Information and communication refer to the procedures used by an organization to locate, gather, and transmit pertinent information within the limits set by management to fulfill its financial reporting purpose [42,50,51]. Sharing pertinent information with all significant organizational departments is another aspect of effective communication [46].

Research shows that creating and implementing internal controls alone does not guarantee their effectiveness unless the control process is regularly monitored to ensure that it operates as intended [49,52]. Therefore, monitoring is a crucial element of the internal control system framework. It helps evaluate the standard of implemented control mechanisms and their effectiveness in addressing identified risks [10,45]. Adegboyegun et al. [42] define monitoring as actions taken to evaluate the system’s effectiveness over time.

Although related to internal control, financial accountability more specifically involves the maintenance and provision of financial records to ensure that an organization complies with applicable laws and ethical standards [6]. At the core of accountability lies answerability, which means that an organization has both the obligation and the ability to fully address questions concerning its decisions and actions [4]. Financial accountability requires institutions to disclose information about their fiscal activities, promoting transparency in fiscal terms both within and beyond the organization and providing a basis for sound management decisions [14]. Organizational financial performance pertains to the financial outcomes of an organization over a specific period [53]. It can be gauged using various financial indicators such as revenue, profit, return on investment, earnings per share, cash flow, among others. A company’s financial performance is an indispensable aspect of assessing its overall health, growth potential, and long-term viability.

Previous literature has explored the relationships between internal control, financial accountability, and financial performance, but the findings have been mixed. Ejoh and Ejom [54], for instance, did not find a significant association between internal control activities and financial performance of the Cross River State College of Education in Nigeria based on questionnaires and document review. On the other hand, Buallay et al. [55] used return on assets, return on equity, and Tobin’s Q as measures of firm performance and found that a high level of corporate governance, which includes internal control measures, is not linked to enhanced performance for companies listed on the Saudi stock exchange. Oppong et al. [7], meanwhile, investigated the correlation between the level of internal control and the performance of seven faith-based non-governmental organizations (NGOs) in Ghana. Based on self-administered structured questionnaire data, they reported that an internal control system significantly improved the performance of faith-based NGOs, but did not necessarily lead to greater effectiveness. Caplan [56] also maintains that while internal controls can aid in the prevention and detection of errors, they alone are inadequate for preventing management fraud.

Caplan [56] notes that while an internal control framework can aid an organization in achieving its objectives, it may not be adequate for ensuring the organization’s long-term success. However, some studies have established a positive correlation between internal control and financial performance. For instance, risk reporting, which is an aspect of internal control, was shown to enhance the financial performance of Takaful and the cooperative insurance industry in the KSA [57]. Similarly, Umar and Dikko [2] discovered a positive and significant relationship between the performance of commercial banks and various components of internal control, such as control environment, control activities, monitoring, and risk assessment.

Previous research also suggests that internal control and financial accountability are interconnected. According to Widyaningsih [6], internal control and financial accountability work together to promote the overall success of an organization. Kewo [13] found that internal control has a significant influence on the financial accountability of local governments in Indonesia. Similar results were reported for government organizations in New Zealand [23]. Implementing an internal control system can improve financial accountability by enhancing professionalism and minimizing errors in financial statements and reporting [14,24]. However, the impact of internal control on financial accountability can be mixed, as shown in the study by Rafindadi and Olanrewaju [27], which found that an internal control system can either enhance or derail the quality of services rendered by NGOs in Nigeria. Nevertheless, internal controls can help prevent mismanagement of funds and ensure proper accountability, including financial reporting, to stakeholders. NGOs with an operational internal control system in place tend to be more financially stable and are also more likely to grow over time [27].

Literature also shows evidence that a sound financial reporting system can reduce the chances of financial errors and mismanagement, leading to improved financial performance. For instance, Kewo [13] argued that improved financial accountability can lead to better financial performance. Similarly, Wynn-Williams [24] demonstrated that public sector organizations can strengthen financial performance by implementing improved accountability reporting systems with internal and process benchmarking along with increased public documentation. Additionally, a study of 10 Nigerian insurance companies found that risk management, which includes financial accountability, enhances organizational performance and reputation [58]. It is also argued that increased financial accountability can diminish irregularities in financial management, leading to greater trust in the organization among funders and other stakeholders, and subsequently improving the overall financial performance of the organization.

Research has shown that the components of internal control have different effects on the financial accountability of organizations. Widyaningsih [6] found that the control environment, control activity, and supervision significantly affect financial accountability, while risk assessment and information and communication have no such effect. However, all aspects of internal control simultaneously exert a significant effect on financial accountability [6]. Kewo and Afiah [12] also found that an internal control system and internal audit have a positive effect on the quality of financial statements. Similarly, Miah and Mia [23] argued that appropriate accounting control systems are necessary for the relationship between a decentralized structure and district office performance.

As shown above, the literature has reported mixed results on the relationships between internal control, financial accountability, and financial performance, which calls for further empirical investigation. This is particularly relevant for a high-income country like the KSA, where healthcare expenditure is increasing and impacting the national budget. Although the private sector in KSA is still developing, it has the potential to provide alternative healthcare delivery options, which can alleviate the negative effects of the overburdened public healthcare system. Therefore, it is important to explore how internal control is related to financial accountability and financial performance in this context. Drawing on the literature review, this study posits the following hypotheses:

- Hypothesis 1 (H1): Internal control influence the financial performance of private hospitals in the KSA.
- Hypothesis 2 (H2): Internal control influence the financial accountability of private hospitals in the KSA.
- Hypothesis 3 (H3): Financial accountability influence the financial performance of private hospitals in the KSA.
- Hypothesis 4 (H4): Financial accountability mediates the influence of internal control on the financial performance of private hospitals in the KSA.

## Material and methods

### Study design and sample

This study was based on a cross-sectional survey design with a quantitative approach to data collection and analysis to determine the relationship between the internal control components, financial accountability, and financial performance of private healthcare providers in the KSA. Data were collected online using a self-reported questionnaire designed for and administered to private hospitals in the KSA, using SurveyMonkey. A link to the survey was distributed to respondents via WhatsApp groups.

We developed the questionnaire in both English and Arabic with the questions refined through a review process sent to professionals representing the target population who critiqued the questions and provided feedback. The questionnaire was initially designed in English and then translated into Arabic. Two bilingual experts in English and Arabic translated the questionnaires, and we used the back-to-back translation method until the two versions converged [59]. The questionnaire’s content validity was evaluated through both face validity and a pilot study. To establish face validity, academics with extensive expertise in questionnaire design were consulted. They reviewed the questionnaire’s content and suggested changes related to language and phrasing. The experts approved the questionnaire’s content after these revisions were made. Through this process, the questionnaire’s face validity was confirmed.

In addition, a pilot survey was conducted between August 2021 and September 2021 with 24 respondents who were not part of the sample used for model analysis. The pilot study indicated that all scales were reliable, as indicated by Cronbach’s alpha coefficient values above the recommended threshold of 0.7. Pearson correlations were also used to check for internal consistency and found that all items were significantly correlated with the proposed dimension, with positive correlations greater than 0.2, indicating an acceptable level of consistency for the survey instrument. The questionnaire was distributed to prospective participants for data collection from October 2021 to February 2022. The data were collected based on a 5-point Likert-type scale questionnaire from a sample of 102 questionnaires. Of the 102 questionnaires, 78 were included in the final sample for analysis. The 24 questionnaires excluded from the analysis were dropped for the following reasons: six were dropped due to missing assessments in two whole variables and 12 questionnaires were not from the hospital category. Among the remaining 84 questionnaires, we detected regular responses by examining the standard deviation values for each questionnaire, which led us to exclude 5 more questionnaires from the final sample. Finally, one questionnaire response was dropped as an outlier in the regression model with a Cook’s distance score greater than 0.1, thereby exceeding the threshold proposed by Weinberg and Abramowitz [60]. Through this process, we arrived at the final dataset of 78 questionnaire responses for our analysis.

### Variables and analysis methods

**Fig 1:**
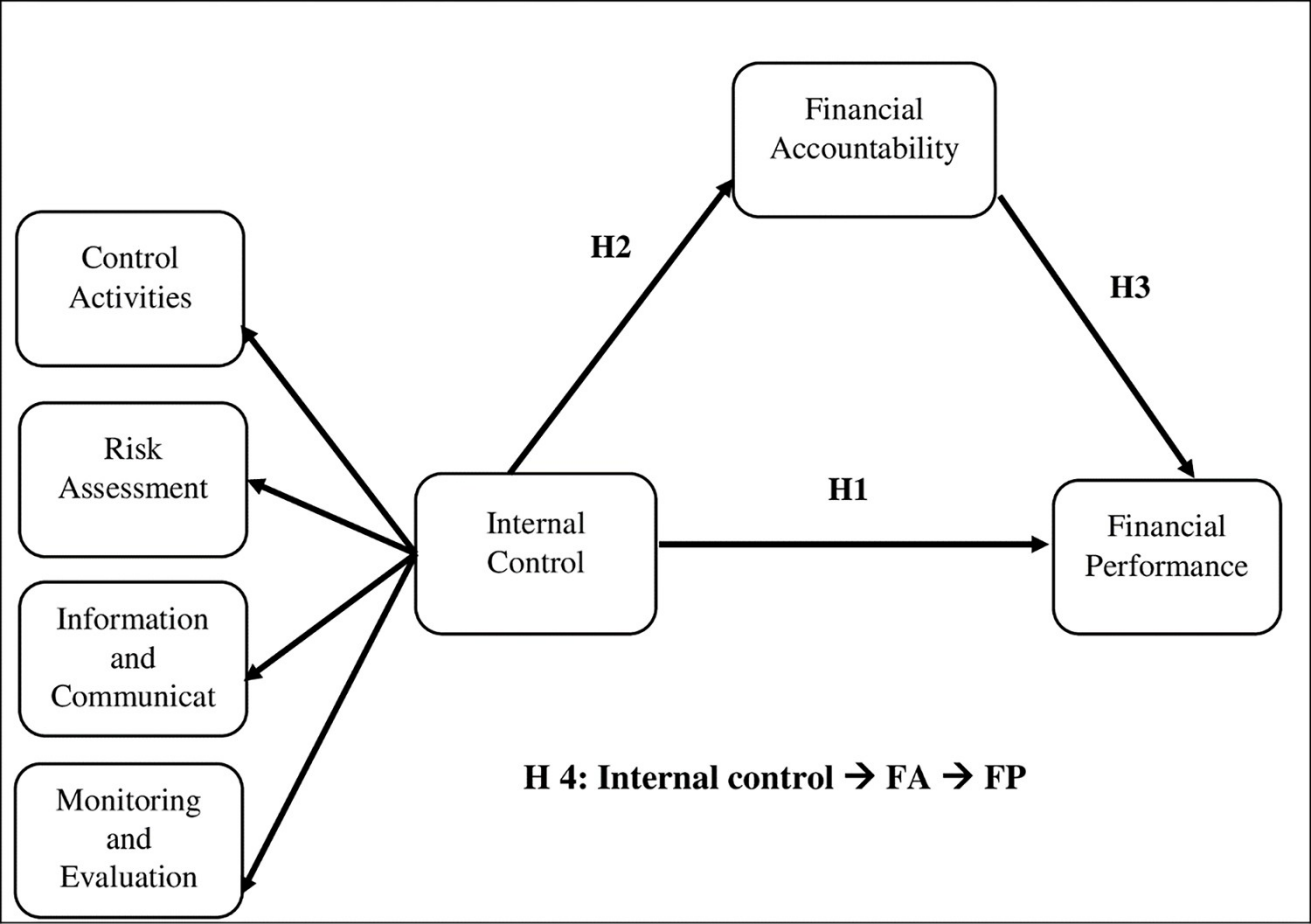
Relationships between internal controls, financial accountability, and financial performance.

To determine the relationships between the internal control components, financial accountability, and financial performance of private healthcare providers in the KSA, we developed the conceptual framework shown in Fig 1, adapted from various studies [61–63], involves three variables: internal control as the independent variable, financial performance as the dependent variable, and financial accountability as the mediating variable. Internal control is a systematic process that encompasses control activities, risk assessment, information and communication, and monitoring and evaluation. It has a pervasive impact on all aspects of an organization’s operations, including administrative, financial, and accounting activities.

We first examined pre-assumptions of the model in relation to the dataset. First, the data were checked for skewness and kurtosis values not exceeding ±2.2, which are considered to demonstrate that the data meet the assumptions of a normal distribution [64]. We further explored potential bias using Harman’s one-factor test, in which the first factor identified should explain no more than 50% of the total variance [65]. Finally, to assess multi-collinearity issues among the factors of concern, we calculated the variance inflation factor (VIF), tolerances, and Pearson correlation coefficients. All components showing correlations with each other should be at the 0.01 level; Pearson correlation coefficients should be below r = 0.90; the VIF values should be below 10; and the tolerance values should be above 0.01, in accordance with Pallant’s [66] recommendations for assessing multicollinearity. Later in the study, the findings for the multicollinearity tests are presented.

PLS-SEM was used to examine the measurement model and estimate the structural model. We selected PLS-SEM as this multivariate approach is widely used to estimate path models comprising latent constructs [67]. Moreover, PLS-SEM is considered to be better suited to handle assumption violations such as normality and sample size compared with covariance-based SEM [67,68]. In brief, we used CFA to identify a set of observables to represent the relationships examined in the proposed extended model. SEM was then used to test the four central hypotheses with both internal controls and financial accountability posited as significant predictors of financial performance.

Therefore, the structural models were assessed through CFA. The measurement model was then used to validate the factor structure by examining the validity and reliability of the measures. This was accomplished by assessing the internal consistency reliability of the indicators, including factor loading (FL) (with a minimum threshold of 0.70) [69], construct reliability, and internal consistency, using Cronbach alpha (minimum threshold of 0.70), and composite reliability (CR) coefficients (minimum threshold of 0.70) [69,70]. The extent to which the measure indicators closely reflect the same theoretical concept was determined through average variance extracted (AVE) coefficients to assess convergent validity (minimum AVE threshold of 0.50) [69]. The results of the CFA are reported later in the study.

Discriminant validity, which assesses the distinctiveness of each construct from other constructs in the model, was determined using the Fornell and Larcker method [71] and the Heterotrait-Monotrait (HTMT) ratio of correlations approach. The Fornell-Larcker criterion compares the square root of the AVE with the correlation of latent constructs. The square root of each construct’s AVE should be greater than its correlation with other latent constructs to demonstrate discriminant validity. An HTMT ratio of the correlation close to 1 suggests a lack of discriminant validity, as proposed by Henseler et al. [72]. Finally, the structural model was evaluated to determine the relationships between the variables by conducting a path analysis, providing insights to evaluate the tested hypotheses.

### Ethical considerations

All procedures performed in this study involving human participants complied with the institutional and/or national research committee ethical standards, and the 1964 Helsinki declaration and subsequent amendments or equivalent ethical standards. This research has been reviewed and given a favourable opinion by King Abdulaziz University. The study was designed and conducted in accordance with the ethical principles established by King Abdulaziz University. Therefore, ethical approval was obtained from the Biomedical Ethics Research Committee, Faculty of Medicine, King Abdulaziz University (Ref-02-21). Online informed consent were obtained from all participants before proceeding with the questionnaire.

## Results

### Demographic and descriptive statistics of the sample

The respondent demographics are presented in Table 1. Significantly more of the respondents were male (n = 70, 89.7%) than female (n = 8, 10.3%). Most of the respondents were young, according to the following breakdown for the three largest age groups: 40–49 years (n = 33, 42.3%), 30–39 years (n = 24, 30.8%), and 50–59 years (n = 13, 16.7%). The youngest respondents (18–29 years) and the oldest respondents (60 years and older) constituted the smallest groups (n = 4, 5.1% for each category). The majority of respondents had high educational levels, with the bachelor’s level forming the largest group for the response of highest educational level (n = 50, 64.1%), followed by the master’s level (n = 23, 29.5%). The smallest groups of highest educational level were post-secondary diploma (n = 3, 3.8%) and doctoral level (n = 2, 2.6%). In terms of the professional background in the context of healthcare delivery, the respondents all indicated having adequate relevant experience: respondents with more than 10 years of experience constituted the largest group (n = 31, 39.7%), followed by those with 6–10 years (n = 24, 30.8%), 1–5 years (n = 20, 25.6%), and a very small group with less than 1 year (n = 3, 3.8%) of relevant experience. In terms of the hospital settings in which the respondents held positions, more than half were employed at small hospitals with fewer than 50 beds (n = 46, 59%), although a large proportion were employed at large hospitals with more than 100 beds (n = 28, 35.9%). A small proportion of respondents were employed at hospitals with 51–100 beds (n = 4, 5.1%). Finally, all of the hospitals represented in the sample were located in cities, with the majority in Mecca (n = 55, 70.5%).

**Table 1:** Demographic characteristics of respondents (n = 78).

Characteristic	Sub-group	n	%
Healthcare institution	Hospital with <50 beds	46	59%
	Hospital with 51–100 beds	4	5.1%
	Hospital with >100 beds	28	35.9%
Gender	Male	70	89.7%
	Female	8	10.3%
Age	18–29 years	4	5.1%
	30–39 years	24	30.8%
	40–49 years	33	42.3%
	50–59 years	13	16.7%
	60 years or older	4	5.1%
Education	Post-secondary diploma	3	3.8%
	University degree	50	64.1%
	Master degree	23	29.5%
	Doctoral degree	2	2.6%
Working years	Less than 1 year	3	3.8%
	1–5 years	20	25.6%
	6–10 years	24	30.8%
	More than 10 years	31	39.7%
Total		78	100%

### Measures and validation

The reliability of the data for subsequent analysis was validated. The normal distribution of the data was determined based on two criteria: skewness and kurtosis. All values for the factors of concern in the structural model (i.e., control activities, risk assessment, information and communication, monitoring and evaluation, financial performance, and financial accountability) were within the range of ±2.2, indicating that normality issues were not a concern.

The bias of the dataset was checked using Harman’s one-factor test, showing a cumulative variance below the 50% level (i.e., 40.91%), thereby demonstrating that bias was not a concern. Finally, multicollinearity was not evident among the internal control components: all components were correlated at the 0.01 level; correlation coefficients were below r = 0.90; VIF values were below 10; and the tolerance values were above 0.01, in conformance with Pallant’s recommendation for the multicollinearity check (Table 2).

**Table 2:** Multicollinearity evaluation among the internal control components (n = 78).

Factor	Tolerance	VIF	Pearson correlation coefficient			
			CA	RA	IC	ME
Control activities (CA)	0.472	2.118	1			
Risk assessment (RA)	0.549	1.823	0.659***	1		
Information and communication (IC)	0.630	1.586	0.592***	0.495***	1	
Monitoring and evaluation (ME)	0.407	2.459	0.727***	0.670***	0.610***	1

*** Correlation is significant at the 0.01 level.

### Confirmatory factor analysis

The findings for CFA are presented in Table 3. The psychometric properties of the constructs in our measurement model were then validated using Smart PLS.

**Table 3:** Indicator reliability, construct reliability, and convergent validity (n = 78).

Construct	Indicator	AVE	Cronbach’s alpha	CR	FL
Control activities (CA)	CA1: Accounts are reconciled on a monthly basis to uncover errors and prevent fraud	0.645	0.729	0.844	0.796***
	CA4: All financial transactions are recorded in vouchers for future references				0.740***
	CA5: This healthcare provider undergoes regular audits				0.868***
Risk assessment (RA)	RA1: Mechanisms have been established to detect and respond to changes that could have a significant impact on the healthcare provider	0.706	0.861	0.905	0.730***
	RA2: Risks are evaluated in connection to changes in the operational environment				0.869***
	RA3: This healthcare provider is dedicated to identifying risks				0.887***
	RA4: All risks facing this healthcare provider are assessed				0.867***
Information communication (IC)	IC2: Staff members have knowledge of internal controls and accountability	0.773	0.853	0.910	0.902***
	IC3: The healthcare provider has well-defined communication channels				0.921***
	IC4: The current flow of information is fast and efficient				0.810***
Monitoring and evaluation (ME)	ME1: Monitoring strategies are employed at any point in the monitoring process	0.679	0.843	0.894	0.854***
	ME2: A reporting system is in place for all the activities of this healthcare provider				0.855***
	ME3: The internal audit function operates free from management influence				0.813***
	ME5: There is a clear division of responsibilities between procurement, accounts payable, and disbursement processes				0.772***
Financial accountability (FA)	FA2: This healthcare provider’s team is well-versed in the use and implementation of accounting and financial management systems	0.740	0.929	0.944	0.868***
	FA3: This healthcare provider makes predictions about cash flow				0.890***
	FA4: This healthcare provider regularly predicts the end-of-year revenue and expenses to help in making informed decisions throughout the year				0.880***
	FA5: Monthly reconciliation of all cash accounts is performed by this healthcare provider				0.773***
	FA6: This healthcare provider has an established procedure to assess the appropriateness and accuracy of the financial information received				0.897***
	FA7: Procedures are established by the healthcare provider to fulfil its financial obligations				0.846***
Financial performance (FP)	FP1: This healthcare provider outperforms other healthcare providers with respect to return on investment	0.641	0.888	0.914	0.815***
	FP2: The growth of the market share of this healthcare provider exceeds that of its competitors				0.771***
	FP3: This healthcare provider outperforms other healthcare providers on sales volume				0.700******
	FP4: The growth in return on investment of this healthcare provider exceeds that of its competitors				0.868***
	FP5: This healthcare provider outperforms other healthcare providers on profit margin (net income/sales)				0.843***
	FP6: In general, the competitive position of this healthcare provider is superior to that of its competitors				0.799***

*** P < 0.01.

To validate the constructs, indicator reliability, followed by construct reliability, and then convergent and discriminant validity were confirmed. In regard to indicator reliability, the FL for each indicator was examined. Following the minimum threshold for FL, the following indicators, all with FL below 0.70, were dropped: CA2, CA3, CA6, FA1, IC1, and ME4 (CA2: Prior to payment, a responsible officer must authorize all payments; CA3: All transactions follow proper payment procedures; CA6: The internal auditor operates independently; FA1: To gain a deeper comprehension of our finances, this healthcare provider produces financial statements that compare budget versus actual; IC1: Information flows freely without obstruction; ME4: During audits, external auditors can utilize the work of internal auditors). Therefore, all of the retained indicators had an FL value higher than 0.70. Further, the t-statistic values were also higher than 1.96, suggesting that all of the indicators were reliable (Table 3).

Construct reliability was supported through CR and Cronbach’s alpha values, which were both higher than 0.70, suggesting that the constructs were reliable and internally consistent (Table 3). Moreover, all AVE coefficients were higher than 0.50 (Table 3), suggesting that the convergent validity of the constructs aligned with the suggested criteria. Moreover, the square root of the AVE values was higher than the intercorrelation of each construct with the remaining constructs, thereby meeting the discriminant validity requirements of Fornell and Larcker’s approach (Table 4).

**Table 4:** Discriminant validity using Fornell and Larcker’s approach (n = 78).

	CA	FA	FP	IC	ME	RA
**CA**	**0.803**					
**FA**	0.710	**0.860**				
**FP**	0.520	0.596	**0.801**			
**IC**	0.579	0.709	0.274	**0.879**		
**ME**	0.646	0.592	0.325	0.542	**0.824**	
**RA**	0.625	0.618	0.386	0.438	0.662	**0.840**

Abbreviations: CA, control activities; FA, financial accountability; FP, financial performance; IC, information communication; ME, monitoring and evaluation; RA, risk assessment.

Further, discriminant validity was confirmed through assessment of the HTMT ratio of correlations, which were all below 1 (see Table 5).

**Table 5:** Discriminant validity assessment using the Heterotrait-Monotrait approach (n = 78).

	CA	FA	FP	IC	ME	RA
**CA**						
**FA**	0.848					
**FP**	0.597	0.639				
**IC**	0.732	0.788	0.290			
**ME**	0.822	0.659	0.346	0.624		
**RA**	0.782	0.682	0.407	0.516	0.791	

Abbreviations: CA, control activities; FA, financial accountability; FP, financial performance; IC, information communication; ME, monitoring and evaluation; RA, risk assessment.

Based on the higher-order measurement model established, the internal control components were found to be significantly correlated to the internal control latent variable, and all correlation coefficients were above 0.70. These correlation results support the operationalization of internal control: control activities (Y = 0.840, P < 0.001), risk assessment (Y = 0.849, P < 0.001), information communication (Y = 0.748, P < 0.001), and monitoring and evaluation (Y = 0.880, P < 0.001). To conclude, indicator reliability, construct reliability, and convergent and discriminant validity criteria were all met for the model constructs, demonstrating their suitability for PLS-SEM.

### PLS-SEM results

As shown in Table 6, based on the respondents’ assessments, private hospitals in the KSA appear to practice internal control processes at a high level (mean = 3.73). Financial accountability also showed a high score (mean = 3.69), whereas financial performance was more moderate (mean = 3.31). Agreement was evident among the respondents given that none of the standard deviation values was higher than 1. Finally, Pearson correlations provided insights relative to the correlations between the factors of concern. Specifically, we found initial support for our model prepositions, with all factors showing significant correlations to each other at the 0.01 level.

**Table 6:** Descriptive statistics of the partial least-squares-structural equation model (n = 78).

Factor	Mean	Level+	Standard deviation	Min	Max	Pearson correlation		
						Internal controls	FA	FP
Internal controls	3.73	High	0.61	2.23	5.00	1		
FA	3.69	High	0.75	1.00	5.00	0.788***	1	
FP	3.31	Moderate	0.60	1.67	4.83	0.418***	0.588***	1

*** Correlation is significant at the 0.01 level.

+ The mean values were interpreted based on the scale proposed by Sekaran and Bougie [70] and adopted by previous studies (e.g., Al Rahahleh [73]) as follows: 3.67–5.00, high level of agreement; 2.34–3.669, moderate level of agreement; 1–2.339, low level of agreement.

Abbreviations: FA, financial accountability; FP, financial performance.

**Fig 2:**
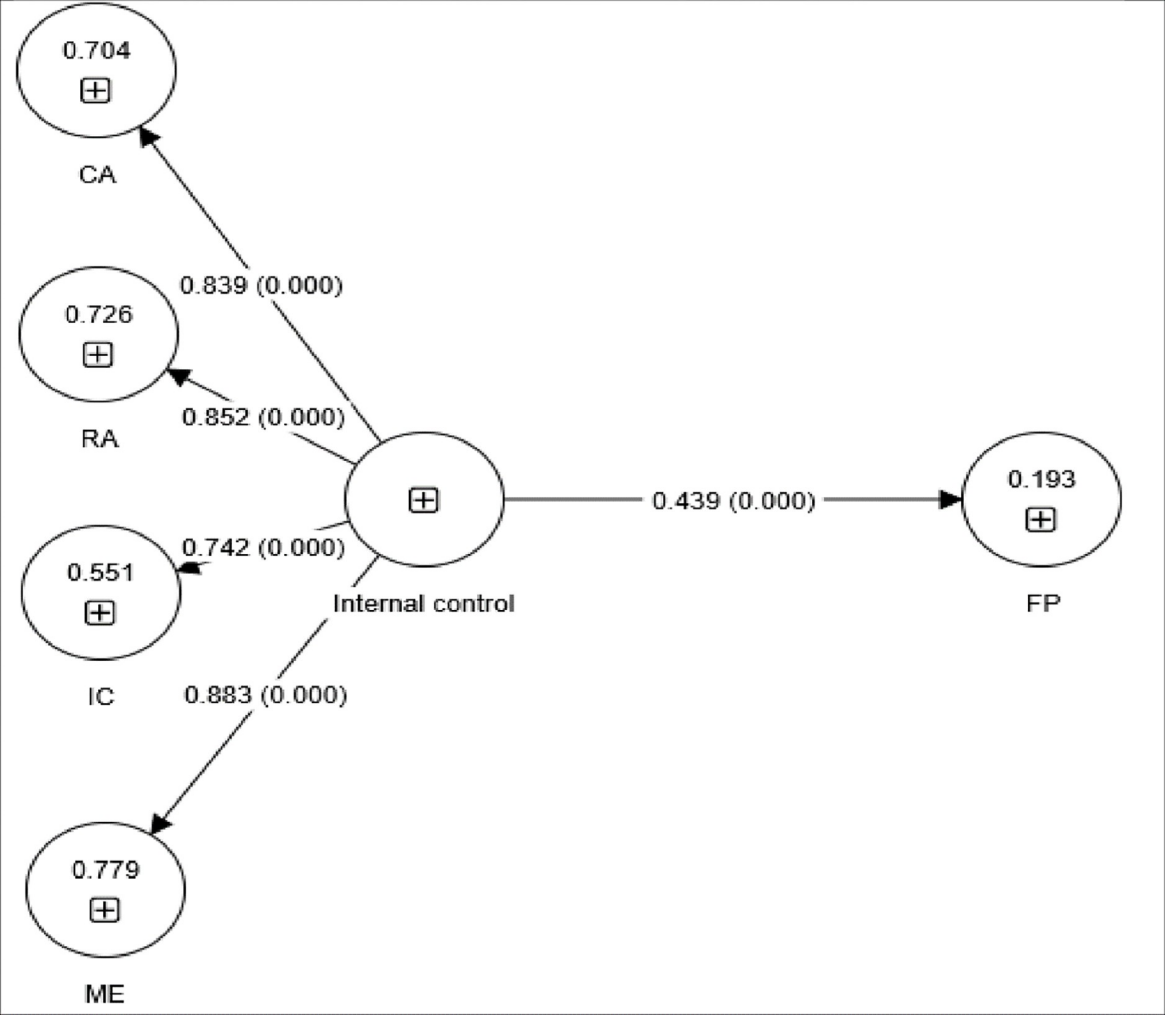
Structural model used to test the direct influence of internal control components on financial performance (FP). CA, control activities; IC, information communication; ME, monitoring and evaluation; RA, risk assessment.

We used PLS-SEM to examine the structural models, establishing three models to test our hypotheses. Fig 2 shows the structural model tested for the direct influence of internal control on financial performance. The coefficient of determination (R^2^) was 19.3%, indicating that internal control explained an acceptable level of the variance in financial performance. Internal control was found to have a significant positive influence on financial performance (β = 0.439, P = 0.000); namely, for every 1% increase in internal control, financial performance improved by 43.9%. Therefore, H1 was supported.

**Fig 3:**
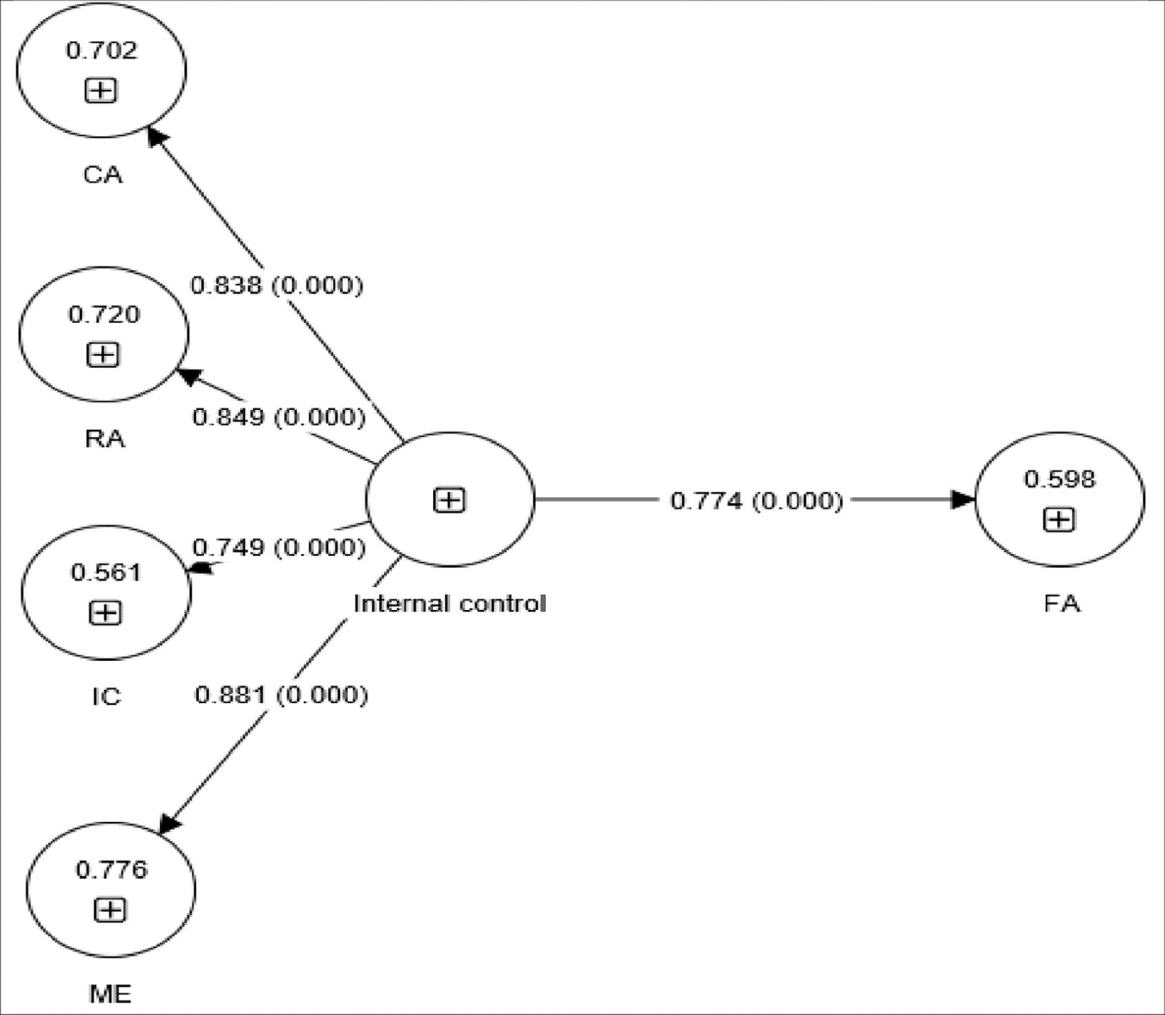
Structural model testing for the direct influence of internal control components on financial accountability (FA). CA, control activities; IC, information communication; ME, monitoring and evaluation; RA, risk assessment.

Fig 3 presents a structural model showing the significant influence of internal control on financial accountability (β = 0.774, P = 0.000). Namely, for every 1% increase in internal control, financial accountability improved by 77.4%. Therefore, H2 was supported.

**Fig 4:**
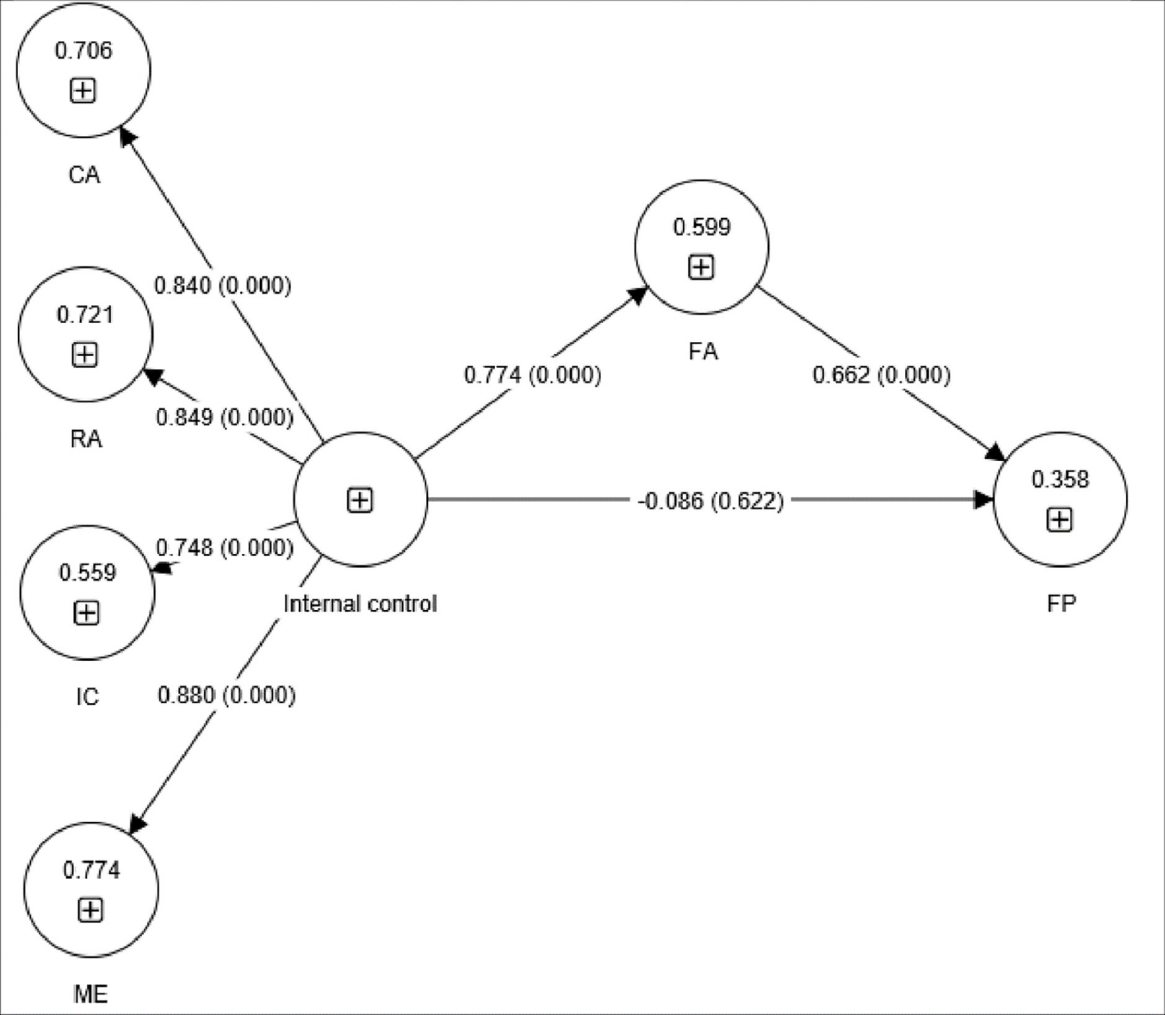
Structural model testing for financial accountability (FA) as a mediator between internal control and financial performance (FP). CA, control activities; IC, information communication; ME, monitoring and evaluation; RA, risk assessment.

Fig 4 presents a structural model with financial accountability introduced as a mediator between internal control and financial performance. The coefficient of determination (R^2^) in financial accountability was 59.9%, indicating that internal control explains a substantial amount of the variance in financial accountability. Further, the R^2^ in financial performance increased to 35.8%, indicating that financial accountability contributed to financial performance. Moreover, our model exhibited a high level of predictive relevance, as the Q2 values for both financial accountability (Q2 = 0.437) and financial performance (Q2 = 0.208) were higher than 0.15. These findings demonstrated that our model has adequate quality for the investigative purpose. Multicollinearity did not appear to be an issue in any of the models as none of the VIF values was greater than 10.

In terms of the influence of financial accountability on financial performance (β = 0.662, P = 0.000), we found that for every 1% increase in financial accountability, financial performance improved by 66.2%. Therefore, H3 was supported. The indirect influence is the product of H2 × H3 = 0.774 × 0.662 = 0.512, which was significant, with bootstrapping results yielding P = 0.000. In fact, financial accountability was found to be a full mediator between internal control and financial performance: with financial accountability controlled for, the direct influence of internal control on financial performance was non-significant and negative (β = –0.086, P = 0.623). Therefore, H4 was supported. A summary of the hypothesis testing is presented in Table 7.

**Table 7:** Summary of hypothesis testing.

*Hypothesis*	Path	Beta	Decision
***H1***	Internal control → FP	0.439***	Supported
***H2***	Internal control → FA	0.774***	Supported
***H3***	FA → FP	0.662***	Supported
***H4***	Internal control → FA → FP	Direct influence = –0.086; Indirect influence = 0.512***; Total effect = 0.426***	Supported as a full mediator

***P < 0.01.

Abbreviations: FA, financial accountability; FP, financial performance.

## Discussion

This study examined the relationships between internal control, financial accountability, and financial performance in the private healthcare sector in the KSA. The principal contribution of this study to the literature lies in its specific application to the private healthcare sector and its focus on financial accountability and financial performance. These represent points of departure from the research in the field to date, given the previous emphasis on internal control relative to quality-of-service delivery in public and private settings [24,29,30]. This study contributes to the related literature on the healthcare sector in the KSA, where there is an increasing interest in involving the private sector in healthcare delivery. As limited evidence exists regarding the relationships between internal control, financial accountability, and financial performance in the healthcare system, this study fills this gap and provides evidence-based policy recommendations not only for the KSA but also for other countries with similar socio-economic characteristics, particularly emerging economies.

The study utilized various analytical methods to mitigate the risk of generating results that are solely driven by the chosen methodology. The study applied both CFA and SEM using PLS-SEM approach. These techniques were employed to ensure that the outcomes and discoveries derived from the study are dependable and resilient. Consequently, the study can provide a solid foundation for implementing internal control and financial accountability measures that could enhance the financial performance of private hospitals in the KSA.

The descriptive statistics indicate that the private hospitals in the KSA sampled have adequate internal control practices (mean = 3.73) and a satisfactory level of financial accountability (mean = 3.69), whereas only a moderate level of financial performance was identified (mean = 3.31), indicating that specific steps should be taken to improve this latter sphere. This finding is in line with Oppong et al.’s [7] observation that internal control helps an organization to achieve its goals and objectives, but does not necessarily improve effectiveness. As Caplan [56] argued, internal controls can be useful in preventing and detecting errors, but are insufficient in terms of preventing management fraud. Therefore, to ensure strong financial performance, management must have the desire and the discipline to implement sound financial practices.

The finding that for every 1% increase in internal control, financial performance improved by 43.9%, with R^2^ = 19.3%, supports H1 that internal control positively and significantly influences financial performance. This result contradicts with some of the findings presented in the literature [54,55], but aligns with the dominant finding that internal control exerts a significant influence on financial performance [1,2,5,26,57]. This is likely the case because internal control ensures that an organization’s objectives are being achieved. Poor internal controls often enable fraudulent activity to go unchecked, which will almost inevitably result in an organization’s downfall in the long-term. Any organization faces numerous risks and problems when effective internal control mechanisms are not in operation, and the healthcare sector may be uniquely vulnerable given its composition of employees with divergent professional and non-professional job skills and responsibilities who face unpredictable demands and outcomes [24] in a stressful environment. The findings of the present study thereby underscore the importance of internal control as essential to minimizing errors and strengthening performance in such an environment.

This study also found that for every 1% increase in internal control, financial accountability improved by 77.4%, with R^2^ = 59.9%. This result indicates that internal control substantially accounts for financial accountability, thereby supporting H2. Similar findings have been reported in several previous studies [6,13,14,27,30]. An effective internal control system improves accountability and financial reporting [27]. Based on the sample data, the high level of internal control in the private hospitals of the KSA should mean that financial reports and other necessary documents can be easily generated and are readily available to interested parties. Well-functioning internal control systems ensure that fraudulent activities and fiscal mismanagement are minimized, thereby increasing the chances that a hospital will be able to account for its actions and decisions in a timely, comprehensive, and accurate manner.

In terms of the direct influence of financial accountability on financial performance, we found that for every 1% increase in financial accountability, financial performance improved by 66.2%, with R^2^ = 35.8%. On this basis, H3—financial accountability influences financial performance—was supported. According to Sari et al. [25], accountability constitutes the foundation for the proper functioning of any organization focused on service delivery. However, Al-Matari et al. [74] failed to establish a relationship between financial accountability (defined as action taken by the board of directors and audit committees) and the performance of firms in the KSA. Sharma and Senan [29] stressed the existence of significant differences in the effectiveness of selected Saudi banks depending on their internal control and financial accountability levels. Organizations that operate at a high level of accountability ensure the efficient and effective use of resources in line with given objectives and targets [4].

Finally, this study established that financial accountability acts as a full mediator between internal control and financial performance, given that the direct influence of internal control on financial performance was identified as non-significant and negative. Accordingly, H4—financial accountability mediates the influence of internal control on financial performance—was supported. This is not surprising, as internal control and financial accountability work hand-in-hand to support and advance organizational performance. Miah and Mia [23] showed that the relationship between decentralization and district office performance relies on the mediating role of the accounting control system. Similarly, internal control is most effective when supported by and integrated with a strong financial accountability system. An excellent control system without proper financial accountability may have little impact on financial performance. It is the accountability aspect that multiplies the effect of internal control on financial performance [29].

In sum, the study’s implications for private healthcare organizations in KSA are significant. Firstly, these organizations should implement specific measures to improve their financial performance by adopting sound financial practices. Secondly, the study underscores the importance of internal control measures in reducing errors and enhancing performance within the private healthcare industry’s stressful and unpredictable setting. Thirdly, the study recommends that private healthcare organizations put in place efficient internal control systems to prevent fraudulent activities and mishandling of funds, which will enhance their financial reporting and accountability. Fourthly, the study highlights that prioritizing accountability is essential for private healthcare organizations to improve their financial performance by ensuring the effective and efficient utilization of resources to achieve their goals and objectives. Hence, this study stands out for its emphasis on financial accountability and financial performance in the private healthcare sector. The study’s application of multiple analysis techniques provides valuable insights for improving the financial performance of private hospitals in the KSA through implementing internal control and financial accountability measures.

Nevertheless, the study has some limitations. Firstly, it is limited in its generalizability as it only focuses on private hospitals in the KSA and relies on self-reported data, which could introduce bias. Secondly, the study does not control for external factors that may influence financial performance, such as changes in the regulatory environment, economic conditions, or competitive landscape. Lastly, it may not fully account for the potential bidirectional relationship between internal control, financial accountability, and financial performance, which future studies could explore using alternative techniques. Further research can build upon this study by exploring how additional factors, such as healthcare regulations and policies, human resource management, consumer confidence, and new technologies, may act as mediators or moderators to improve the financial performance of healthcare organizations. As demand for healthcare services continues to rise in the KSA, it is crucial to establish effective internal control and financial accounting systems in the private healthcare sector, aligning with the objectives of the Kingdom’s Vision 2030.

## Conclusion

In this study, we utilized a questionnaire instrument to collect data and employed CFA as well as PLS-SEM techniques to investigate the relationships between internal control, financial accountability, and financial performance in the private healthcare sector in the KSA. Our results showed that while there was a high level of internal control and financial accountability in this context, financial performance was only moderate. Additionally, we found that financial accountability had a direct influence on financial performance. However, our findings suggest that financial accountability serves as a mediating mechanism through which internal control significantly affects the operational effectiveness of private hospitals in the KSA, thus promoting financial performance. Based on the results of the study, policy recommendations for private hospitals in the KSA could include the implementation and monitoring of appropriate internal control and financial accountability systems. This may involve ensuring that hospitals have robust internal control environments, control activities, and supervision mechanisms in place. Additionally, hospitals may need to improve their risk assessment and information and communication processes to enhance financial accountability. Furthermore, directors and decision-makers may need to ensure that financial accountability is prioritized and that staff members are trained on the importance of financial management and accountability. By taking these steps, hospitals can improve their operational effectiveness and financial performance, ultimately contributing to the overall improvement of the healthcare sector in the KSA and other related countries.

## References

[1] Al-Thuneibat AA, Al-Rehaily AS, Basodan YA. The impact of internal control requirements on profitability of Saudi shareholding companies. International Journal of Commerce and Management. 2015;25(2):196–217.

[2] Umar H, Dikko MU. The effect of internal control on performance of commercial banks in Nigeria. International Journal of Management Research. 2018;8(6):13–32.

[3] Casagrande M, Favieri F, Tambelli R, Forte G. The enemy who sealed the world: effects quarantine due to the COVID-19 on sleep quality, anxiety, and psychological distress in the Italian population. Sleep medicine. 2020;75:12–20. doi: 10.1016/j.sleep.2020.05.011 32853913 PMC7215153

[4] Schedler A. Conceptualizing Accountability. In: Schedler A, Diamond LJ, Plattner MF, editors. The self-restraining state: power and accountability in new democracies. USA: Lynne Rienner Publishers; 1999. p. 13–28.

[5] Socol A. Internal Banking Control and Audit: A Comparative Approach in the Romanian Banking Sector. Annales Universitatis Apulensis: Series Oeconomica. 2011;13(2):396–403.

[6] Widyaningsih A. The influence of internal control system on the financial accountability of elementary schools in Bandung, Indonesia. Research Journal of Finance and Accounting. 2015;6(24):89–96.

[7] Oppong M, Owiredu A, Abedana V, Asante E. The impact of internal control on the performance of faith-based NGOs in Accra. Research Journal of Finance and Accounting. 2016;7(12):110–25.

[8] Jensen MC, Meckling WH. Theory of the firm: Managerial behavior, agency costs and ownership structure. Journal of financial economics. 1976;3(4):305–60.

[9] Furubotn EG, Richter R. Institutions and economic theory: The contribution of the new institutional economics. Ann Arbor: University of Michigan Press; 2005.

[10] Tetteh LA, Kwarteng A, Aveh FK, Dadzie SA, Asante-Darko D. The impact of internal control systems on corporate performance among listed firms in Ghana: The moderating role of information technology. Journal of African Business. 2022;23(1):104–25.

[11] Ogungbade OI, Olweny T, Oluoch O. Effect of Firm Size on Innovation among Manufacturing Companies in Nigeria. European Journal of Business, Economics and Accountancy. 2017;5(6):1–14.

[12] Kewo CL, Afiah NN. Does quality of financial statement affected by internal control system and internal audit? International Journal of Economics and Financial Issues. 2017;7(2):568–73.

[13] Kewo CL. The influence of internal control implementation and managerial performance on financial accountability local government in Indonesia. International Journal of Economics and Financial Issues. 2017;7(1):293–7.

[14] Coy D, Fischer M, Gordon T. Public accountability: a new paradigm for college and university annual reports. Critical perspectives on accounting. 2001;12(1):1–31.

[15] Al-Hanawi MK, Alsharqi O, Almazrou S, Vaidya K. Healthcare finance in the Kingdom of Saudi Arabia: a qualitative study of householders’ attitudes. Applied health economics and health policy. 2018;16:55–64. doi: 10.1007/s40258-017-0353-7 28933057 PMC5797208

[16] Alkhamis AA. Critical analysis and review of the literature on healthcare privatization and its association with access to medical care in Saudi Arabia. Journal of infection and public health. 2017;10(3):258–68. doi: 10.1016/j.jiph.2017.02.014 28343793

[17] Al-Hanawi MK, Vaidya K, Alsharqi O, Onwujekwe O. Investigating the willingness to pay for a contributory National Health Insurance Scheme in Saudi Arabia: a cross-sectional stated preference approach. Applied health economics and health policy. 2018;16:259–71. doi: 10.1007/s40258-017-0366-2 29307076 PMC5874278

[18] Bawazir A, Al-Surimi K, Suwaidan SD, AlShehri AM, AlFarhan AI, Abolfotouh MA. Capacity and readiness of primary health care centers for implementation of the basic strategy for prevention and control of non-communicable diseases in Saudi Arabia.: A case study from the Ministry of National Guard-Health Affairs, Riyadh, Saudi Arabia. Saudi medical journal. 2019;40(6):614. 31219498 10.15537/smj.2019.6.24164 PMC6778751

[19] Al-Hanawi MK, Alsharqi O, Vaidya K. Willingness to pay for improved public health care services in Saudi Arabia: a contingent valuation study among heads of Saudi households. Health Economics, Policy and Law. 2020;15(1):72–93. doi: 10.1017/S1744133118000191 29862936

[20] Al-Hanawi MK, Qattan AM. An analysis of public-private partnerships and sustainable health care provision in the Kingdom of Saudi Arabia. Health services insights. 2019;12:1178632919859008. doi: 10.1177/1178632919859008 31308685 PMC6613064

[21] Rahman R. The privatization of health care system in Saudi Arabia. Health services insights. 2020;13:1–8. doi: 10.1177/1178632920934497 32636636 PMC7315664

[22] Al-Hanawi MK, Almubark S, Qattan AM, Cenkier A, Kosycarz EA. Barriers to the implementation of public-private partnerships in the healthcare sector in the Kingdom of Saudi Arabia. Plos one. 2020;15(6):e0233802. doi: 10.1371/journal.pone.0233802 32555648 PMC7302438

[23] Miah NZ, Mia L. Decentralization, accounting controls and performance of government organizations: a New Zealand empirical study. Financial Accountability & Management. 1996;12(3):173–90.

[24] Wynn-Williams KL. Performance assessment and benchmarking in the public sector: An example from New Zealand. Benchmarking: An International Journal. 2005;12(5):482–92.

[25] Sari N, Ghozali I, Achmad T. The effect of internal audit and internal control system on public accountability: The emperical study in Indonesia state universities. International Journal of Civil Engineering and Technology. 2017;8(9):157–66.

[26] Gordon OO, Kalenzi A. Internal control and quality service delivery in a public health sector: A case study of a Local Government in Uganda. African Journal of Business Management. 2019;13(16):557–63.

[27] Rafindadi AA, Olanrewaju ZA. The impact of internal control system on the financial accountability of non-governmental organisations in nigeria: Evidence from the structural equation modelling. International Review of Management and Marketing. 2019;9(3):49–63.

[28] Alanazi AS, Liu B, Forster J. The financial performance of Saudi Arabian IPOs. International Journal of Islamic and Middle Eastern Finance and Management. 2011;4(2):146–57.

[29] Sharma RB, Senan N. A study on effectiveness of internal control system in selected banks in saudi Arabia. Asian Journal of Managerial Science. 2019;8(1):41–7.

[30] Al-Shetwi M, Ramadili SM, Chowdury THS, Sori ZM. Impact of internal audit function (IAF) on financial reporting quality (FRQ): Evidence from Saudi Arabia. African Journal of Business Management. 2011;5(27):11189–98.

[31] Sarstedt M, Ringle CM, Smith D, Reams R, Hair JF Jr. Partial least squares structural equation modeling (PLS-SEM): A useful tool for family business researchers. Journal of family business strategy. 2014;5(1):105–15.

[32] Dash G, Paul J. CB-SEM vs PLS-SEM methods for research in social sciences and technology forecasting. Technological Forecasting and Social Change. 2021;173:121092.

[33] Nyumoo AK. Effects of Internal Control on the Financial Performance of Saccos in Meru County: Kenya Methodist University; 2020.

[34] Musah A, Padi A, Okyere B, E. Adenutsi D, Ayariga C. Does corporate governance moderate the relationship between internal control system effectiveness and SMEs financial performance in Ghana? Cogent Business & Management. 2022;9(1):2152159.

[35] El-Mahdy DF, Park MS. Internal control quality and information asymmetry in the secondary loan market. Review of Quantitative Finance and Accounting. 2014;43:683–720.

[36] Agyei-Mensah BK. Accountability and internal control in religious organisations: a study of Methodist church Ghana. African Journal of Accounting, Auditing and Finance. 2016;5(2):95–112.

[37] Guo KH, Eschenbrenner BL. CVS Pharmacy: An instructional case of internal controls for regulatory compliance and IT risks. Journal of Accounting Education. 2018;42:17–26.

[38] PireS V, Guimarães AS. Social control of public expenditures in a multilevel principal-agent approach. Brazilian Journal of Political Economy. 2015;35:878–94.

[39] Monsma K. Rethinking rational choice and agency theory: ranchers and managers in the 19th century. Revista Brasileira de Ciências Sociais. 2000;15(43).

[40] Laufer D. Small business entrepreneurs: A focus on fraud risk and prevention. American Journal of Economics and Business Administration. 2011;3(2):401–4.

[41] Ishumgisa L. Antecedents of internal control in National Social Security Fund (NSSF): Makerere University; 2011.

[42] Adegboyegun AE, Ben-Caleb E, Ademola AO, Oladutire E, Sodeinde GM. Internal control systems and operating performance: Evidence from small and medium enterprises (SMEs) in Ondo state. Asian Economic and Financial Review. 2020;10(4):469–79.

[43] Vu Q, Nga NTT. Does the implementation of internal controls promote firm profitability? Evidence from private Vietnamese small-and medium-sized enterprises (SMEs). Finance Research Letters. 2022;45:102178.

[44] Chalmers K, Hay D, Khlif H. Internal control in accounting research: A review. Journal of Accounting Literature. 2019;42(1):80–103.

[45] Chen H, Yang D, Zhang X, Zhou N. The moderating role of internal control in tax avoidance: Evidence from a COSO-based internal control index in China. The Journal of the American Taxation Association. 2020;42(1):23–55.

[46] Hamdan KH. Applying COSO internal control framework to disaster management Evaluation according to hyogo framework for action (HFA) in Iraq. Muthanna Journal of Administrative and Economic Sciences. 2019;9(2):125–52.

[47] Peterson AN. Differences in internal control weaknesses among varying municipal election policies. Journal of Accounting and Public Policy. 2018;37(3):191–206.

[48] Chiu T, Wang T. The COSO framework in emerging technology environments: An effective in-class exercise on internal control. Journal of Emerging Technologies in Accounting Teaching Notes. 2019;16(2):1–10.

[49] Onumah JM, Kuipo R, Obeng VA. Effectiveness of internal control systems of listed firms in Ghana. Accounting in Africa. 12: Emerald Group Publishing Limited; 2012. p. 31–49.

[50] Frazer L. Does internal control improve the attestation function and by extension assurance services? A Practical Approach. Journal of Accounting and Finance. 2020;20(1):28–38.

[51] Badara M. Impact of the effective internal control system on the internal audit effectiveness at local government level. Journal of social and Development Sciences. 2013;4(1):16–23.

[52] Zhou H, Chen H, Cheng Z. Internal control, corporate life cycle, and firm performance. The Political Economy of Chinese Finance. 17: Emerald Group Publishing Limited; 2016. p. 189–209.

[53] Mawanza W. ’The Mordern Corporation’: A Theoretical Review of the Modern Corporation in Zimbabwe and the Use of Share Option Schemes as a Way of Reducing the Agency Problem in Stockmarket Listed Companies. European Journal of Business and Social Sciences. 2014;3(3):28–38.

[54] Ejoh N, Ejom P. The impact of internal control activities on financial performance of tertiary institutions in Nigeria. Journal of Economics and Sustainable Development. 2014;5(16):133–43.

[55] Buallay A, Hamdan A, Zureigat Q. Corporate governance and firm performance: evidence from Saudi Arabia. Australasian Accounting, Business and Finance Journal. 2017;11(1):78–98.

[56] Caplan D. Internal controls and the detection of management fraud. Journal of Accounting Research. 1999;37(1):101–17.

[57] Hemrit W. Determinants driving Takaful and cooperative insurance financial performance in Saudi Arabia. Journal of Accounting & Organizational Change. 2020;16(1):123–43.

[58] Obalola MA, Akpan T, Abass O. The Relationship between Enterprise Risk Management (ERM) and Organizational Performance: Evidence from Nigerian Insurance Industry. Research Journal of Finance and Accounting. 2014;5(14):152–61.

[59] Brislin RW. A culture general assimilator: Preparation for various types of sojourns. International Journal of Intercultural Relations. 1986;10(2):215–34.

[60] Weinberg SL, Abramowitz SK. Statistics using SPSS: An integrative approach: Cambridge University Press; 2008.

[61] Adams C. Factors influencing corporate social and ethical reporting: moving on from extant theories. Accounting, Auditing & Accountability Journal. 2002;15(2):223–50.

[62] Anthony M. Internal Control: Governance frame work and business risk assessment at Reed Elsevier in Auditing. Journal of practice and Theory. 2004;3(6):39–51.

[63] Adeinat I, Kassim N. Extending the service profit chain: the mediating effect of employee productivity. International Journal of Quality & Reliability Management. 2019;36(5):797–814.

[64] Sposito V, Hand M, Skarpness B. On the efficiency of using the sample kurtosis in selecting optimal lpestimators. Communications in Statistics-simulation and Computation. 1983;12(3):265–72.

[65] MacKenzie SB, Podsakoff PM. Common method bias in marketing: Causes, mechanisms, and procedural remedies. Journal of retailing. 2012;88(4):542–55.

[66] Pallant J. SPSS survival manual: A step by step guide to data analysis using IBM SPSS: Routledge; 2020.

[67] Hair JF, Ringle CM, Sarstedt M. PLS-SEM: Indeed a silver bullet. Journal of Marketing theory and Practice. 2011;19(2):139–52.

[68] Rigdon EE. Choosing PLS path modeling as analytical method in European management research: A realist perspective. European Management Journal. 2016;34(6):598–605.

[69] Hair JF Jr, Hult GTM, Ringle CM, Sarstedt M. A primer on partial least squares structural equation modeling (PLS-SEM): Sage publications; 2021.

[70] Sekaran U, Bougie R. Research Methods for Business: A Skill Building Approach. Jhon Wiley and Sons Ltd: United Kingdom. 2019.

[71] Fornell C, Larcker DF. Evaluating structural equation models with unobservable variables and measurement error. Journal of marketing research. 1981;18(1):39–50.

[72] Henseler J, Ringle CM, Sarstedt M. A new criterion for assessing discriminant validity in variance-based structural equation modeling. Journal of the academy of marketing science. 2015;43:115–35.

[73] Al Rahahleh N. Financial Literacy Levels among Saudi Citizens across Budgeting, Saving, Investment, Debt, and Insurance Dimensions. Journal of Risk and Financial Management. 2022;15(12):582.

[74] Al-Matari YA, Al-Swidi AK, Fadzil FH, Al-Matari EM. Board of directors, audit committee characteristics and the performance of Saudi Arabia listed companies. International Review of Management and Marketing. 2012;2(4):241–51.

