# Correction: The financial performance of private hospitals in Saudi Arabia: An investigation into the role of internal control and financial accountability

**DOI:** 10.1371/journal.pone.0309167

**Published:** 2024-08-15

**Authors:** Naseem Al Rahahleh, Tawfeek A. Al-khyal, Abdullah Daghran Alahmari, Mohammed Khaled Al-Hanawi

The first author’s name is spelled incorrectly. The correct name is: Naseem Al Rahahleh. The correct citation is: Al Rahahleh N, Al-khyal TA, Daghran Alahmari A, Al-Hanawi MK (2023) The financial performance of private hospitals in Saudi Arabia: An investigation into the role of internal control and financial accountability. PLoS ONE 18(5): e0285813. https://doi.org/10.1371/journal.pone.0285813
